# A Vision for the Future of Materials Innovation and How to Fast-Track It with Services

**DOI:** 10.1021/acsphyschemau.4c00009

**Published:** 2024-06-12

**Authors:** Lorenz J. Falling

**Affiliations:** School of Natural Sciences, Technical University Munich, 85748 Munich, Germany

**Keywords:** Materials Science, Energy Materials, Measurement Services, Artificial Intelligence, Digital Twin

## Abstract

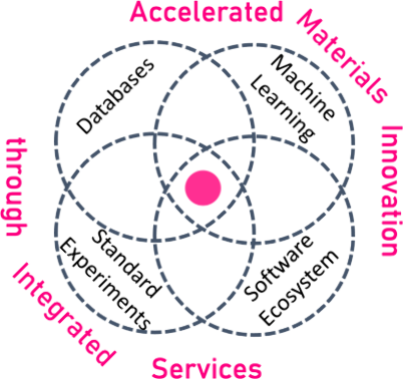

Today, we witness how our scientific ecosystem tries to accommodate a new form of intelligence, artificial intelligence (AI). To make the most of AI in materials science, we need to make the data from computational and laboratory experiments machine-readable, but while that works well for computational experiments, integrating laboratory hardware into a digital workflow seems to be a formidable barrier toward that goal. This paper explores measurement services as a way to lower this barrier. I envision the Entity for Multivariate Material Analysis (EMMA), a centralized service that offers measurement bundles tailored for common research needs. EMMA’s true strength, however, lies in its software ecosystem to treat, simulate, and store the measured data. Its close integration of measurements and their simulation not only produces metadata-rich experimental data but also provides a self-consistent framework that links the sample with a snapshot of its digital twin. If EMMA was to materialize, its database of experimental data connected to digital twins could serve as the fuel for physics-informed machine learning and a trustworthy horizon of expectations for material properties. This drives material innovation since knowing the statistics helps find the exceptional. This is the EMMA approach: fast-tracking material innovation by integrated measurement and software services.

## Where We Might Be Heading

**Figure 1:**
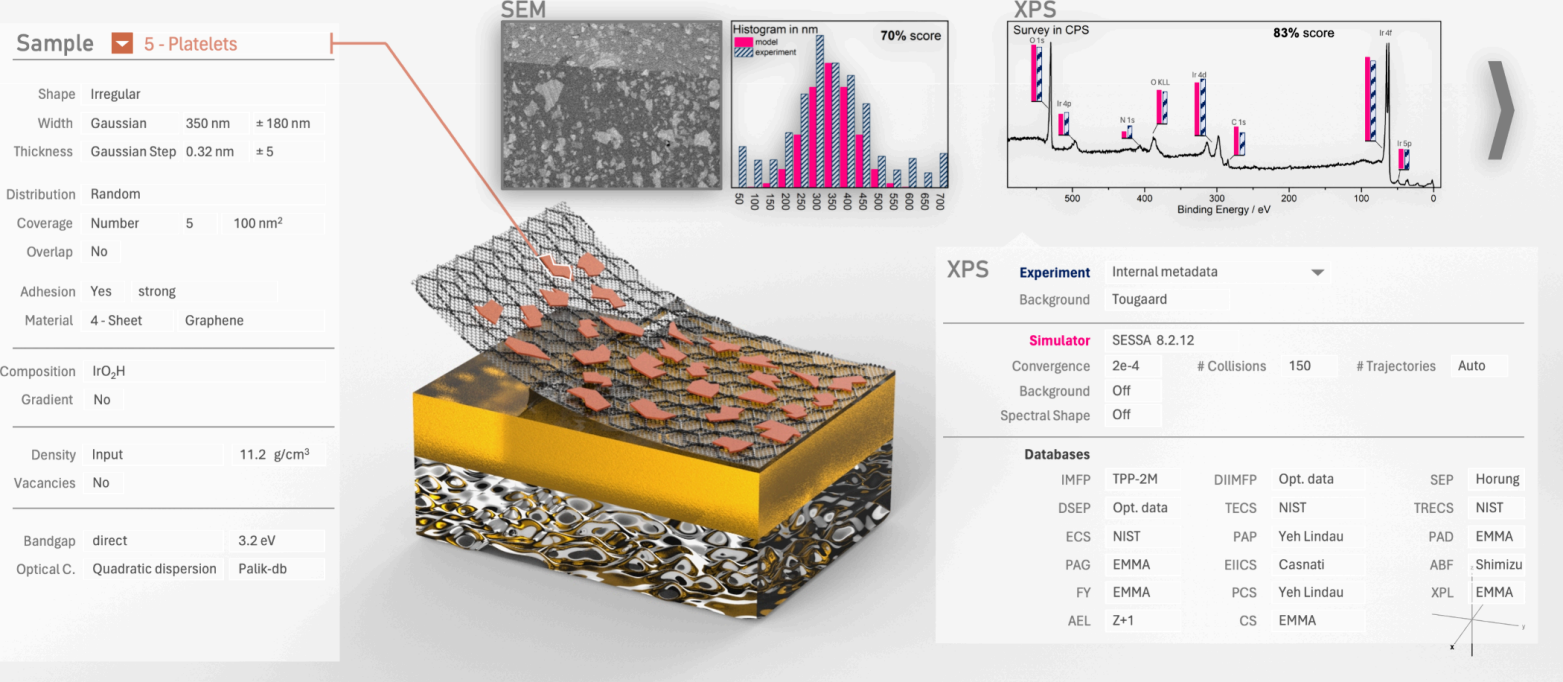
Envisioned user interface of the EMMA; the centralized model is rendered in the center; the sample dialogue on the left shows the properties of the red platelets; two service measurements are shown on top next to their respective results, a scanning electron microscopy image with a histogram of sizes in the center and an X-ray photoelectron spectrum with integrated intensities on the right; experimental results are shown in blue, and the comparison to simulated results is shown in red; the dialogue below the measurement shows the parameters for the spectrum analysis and the simulation of the respective spectrum above it.

*“It is the year 2050. A collaborating student from Abuja spontaneously calls you about a self-healing material for satellite shielding. You activate your glasses and join her in a virtual room of the Entity for Multivariate Material Analysis (EMMA), a measurement service. You see a model representation of her latest sample floating in mid air (*Figure 1*). Upon her visual command, eight graphs reload. Each shows the result of a standardized measurement that was performed at the EMMA. She compares simulated experiments with measured experiments.*

*In an instant, you understand her excitement. The simulations are a close match to the service measurements, and what’s more, the models before and after ion bombardment are almost identical. EMMA’s artificial intelligence (mAI) now suggests an adjustment of the optical constants to improve the match from 89% to 96%. But why should that be the case? You decide to ask SciGpt about a precedent in the literature.*

*During the chat, you come to the conclusion that there is no good reason the optical constants would be as high as mAI suggests. Instead, you wondered if one or a couple of the measurement simulators is not suitable for the sample. After diving into a few documentations of the scattering simulators, you find the issue and pick another simulator for the X-ray scattering measurement. You raise a flag in the mAI suggestion and mark the change as a more reasonable solution to the problem. Your student and you are cautiously satisfied with the 93% match and excited to see the results of the sample after extended ion bombardment. Will your new material hold up?”*

## Where We Are Coming From

Materials science undoubtedly needs artificial intelligence (AI) to uncover the ever more complex relationships between the properties of materials and their function. This need is reflected in multiple initiatives working toward that goal, like the Materials Genome Initiative,^1^ the Materials Innovation Platform,^2^ or the Materials Acceleration Platform.^3^ Their reports agree: data-driven methods could revolutionize materials discovery. We expect to speed up materials discovery by a factor of 10 when fully employing AI in materials research.^4^ This speed-up is desirable and also urgent, considering the systemic importance of materials for the energy transition such as battery cathodes, solar cells, fuel cells, electrolyzers, and catalytic reactors.

Training AI for statistical prediction of materials’ properties requires machine-readable data. As of now, computers learn more efficiently with complete, accurate, and large data sets.^5^ Human researchers are more flexible in that respect. We can learn from the synthesis of heterogeneous data types, including lectures, books, presentations, conversations, figures, tables, databases, etc. The current research system has evolved around this flexible human intelligence and fosters hypothesis-driven, exploratory research. However, uncovering statistical relationships in complex spaces such as the materials space could serve as a horizon of expectation that supports navigating these complex spaces. To make the most of AI in this regard, much of these diverse sources of information need to be made accessible to machines, i.e., machine-readable.

## Where AI Integration Already Thrives

Computational materials science is an early adopter. Projects like NOMAD,^6^ the Materials Project,^7^ Materials Cloud,^8^ AFLOW,^9^ or OQMD^10^–to name the largest–have calculated the formation energies, and other properties, of millions of structures and make them available in a standardized way. Contributions to those databases come from data-conscious researchers, autonomous structure searches, and on-demand computations or services.^11,12^

The large amounts of these FAIR (Findable, Accessible, Interoperable, Reusable)^13^ data sets powered the discovery of more structures and properties predicted by machine learning algorithms. Examples are the Open Catalyst Project,^14^ CrystalLLM,^15^ GNoME,^16^ and MatterGen,^17^ three of which were published last year. The ecosystem is thriving.

However, the current computational costs of ab initio calculations make it impossible to explore the near-infinite space of possible materials and their assemblies. The quantity of structures needs to be weighed against computational accuracy. This is why crystal structures are usually left defect-free, and computational methods are chosen to be cost-efficient at an acceptable error. For the expansion of databases to ever more complex structures, this means that the calculated materials space is either expanded faster at the cost of accuracy or expanded toward more complex structures at the cost of expansion speed.

**Figure 2:**
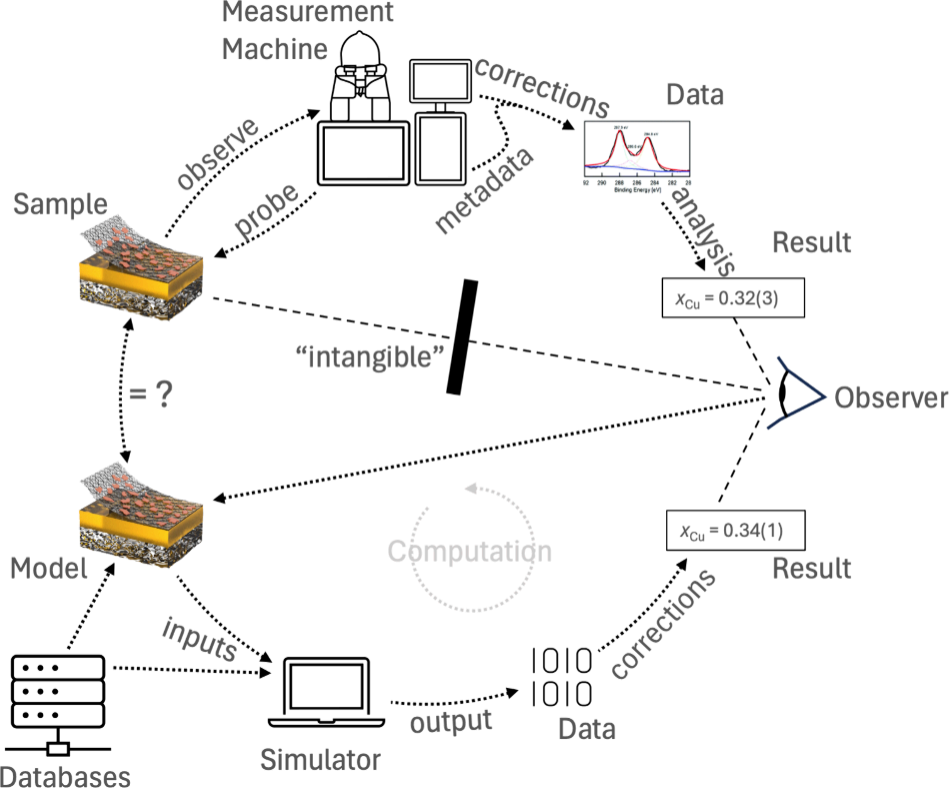
Full cycle of a sample characterization. It starts on the left with a sample of interest; it is “intangible” for the observer, meaning the largest part of its properties are not accessible without measurements; the sample is then probed by a measurement machine, the sample interacts with the probe, and the outcome is observed; the raw data is then corrected using metadata of the machine and further analyzed to obtain a result; if the observer wants to learn from the results, some theoretical or computational prediction is needed, as shown in the lower part of the circle; it starts with a model and a predictive simulator, which can be as simple as “copper came to the surface because no copper was added and the signal spectroscopic intensity got stronger”; if the model and simulator are more complex than that, input from databases is needed to create data; results are obtained from correcting the simulator output data.

Experimental results could aid faster and more accurate computations.^11,18,19^ First, they could deliver benchmarking results to compare to computational results and thereby improve the accuracy of fast computational methods (result-comparison in Figure 2). Second, they could be used to gauge the importance of complexity. Experiments capture the full complexity of materials, including vacancies, dislocations, impurities, adventitious carbon, etc., and they would show how much the properties of materials are influenced by these factors in comparison to computational results (model vs sample in Figure 2).

So what exactly does computational materials science get right and can we repeat that in experimental materials science?

## What It Takes to Make Lab Experiments Machine-Readable

Regardless of whether an experiment is conducted on a computer or in a lab, the basic processes are very similar (compare the top and bottom half in Figure 2). Both have the goal to reduce uncertainty and express the result as a quantity.^20^ This uncertainty can originate from any part of the process chain from a model/sample to the final result (Figure 2), and overall uncertainty is dominated by the weakest link in the chain. To reduce overall uncertainty, it is necessary to record data inputs and data manipulation methods in every step as metadata, a process called data provenance.

Computation is at an advantage here. Since it is inherently digital, it has an input file, a versioned code as a simulator, and an output file (lower half Figure 2). The output is then further corrected/postprocessed to obtain a measure of interest—for example the energy of formation; although the postprocessing still needs more integration,^12^ the digital framework of computation makes it easier to collect all necessary metadata compared to experiments, and it is already being realized for computational experiments in projects like AiiDA^21^ or Globus^22^ and started to expand to experiments.^23,24^

So, the decisive difference between computational and lab experiments is in fact its hardware infrastructure. Computational hardware is generally more compatible and exchangeable than lab hardware, which is built by experimentalists or firms to fit specific research needs. Integrating experimental hardware into digital control and data flow is associated with a software interface and hardware upgrades. Both of these upgrades are necessary to produce machine-readable data, but they are a cumbersome barrier of entry for research groups.

Another advantage for computational experiments is the perfectly controllable model of a manageable size. It brings two advantages. First, models are easier to manipulate than samples, and second, the model is intuitively more accessible than the usually complex experimental samples (Figure 2). An accessible model helps faster learning.

In short, to repeat the success of computational materials science for lab experiments, we have to make lab experiments easy to integrate, explainable with metadata and models, and reported FAIR-ly (Table 1). For further information and viewpoints, the reader is referred to the publications of Jain et al.,^25^ Stein et al.,^26^ Himanen et al.,^11^ and Scheffler et al.^27^

**Table 1:**
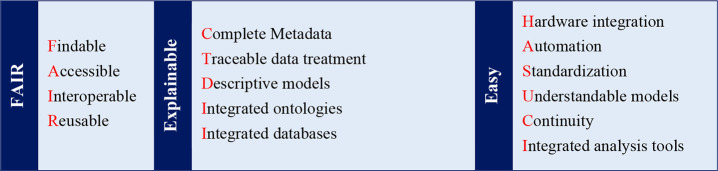
How to Make Experiments Machine-Readable and Accelerate Data-Driven Discovery^a^

a The data needs to be FAIR (left column),^13^ explainable (middle column), and easy to obtain (right column); FAIRness assures long-lasting value of data; explainability reduces uncertainty within the cycle of sample characterization (Figure 2); obtaining this FAIR and explainable data needs to be as easy as possible; i.e., the processes listed in the right column need to have a low barrier.

Reaching the goals in Table 1 will save materials scientists time because, first, FAIR data is easier to reproduce, second, integrated and automated hardware allows higher throughput, and third, descriptive models and accessible analysis tools would make for faster learning.

## How We Are Fostering Machine-Readable Data for Experiments

**Figure 3:**
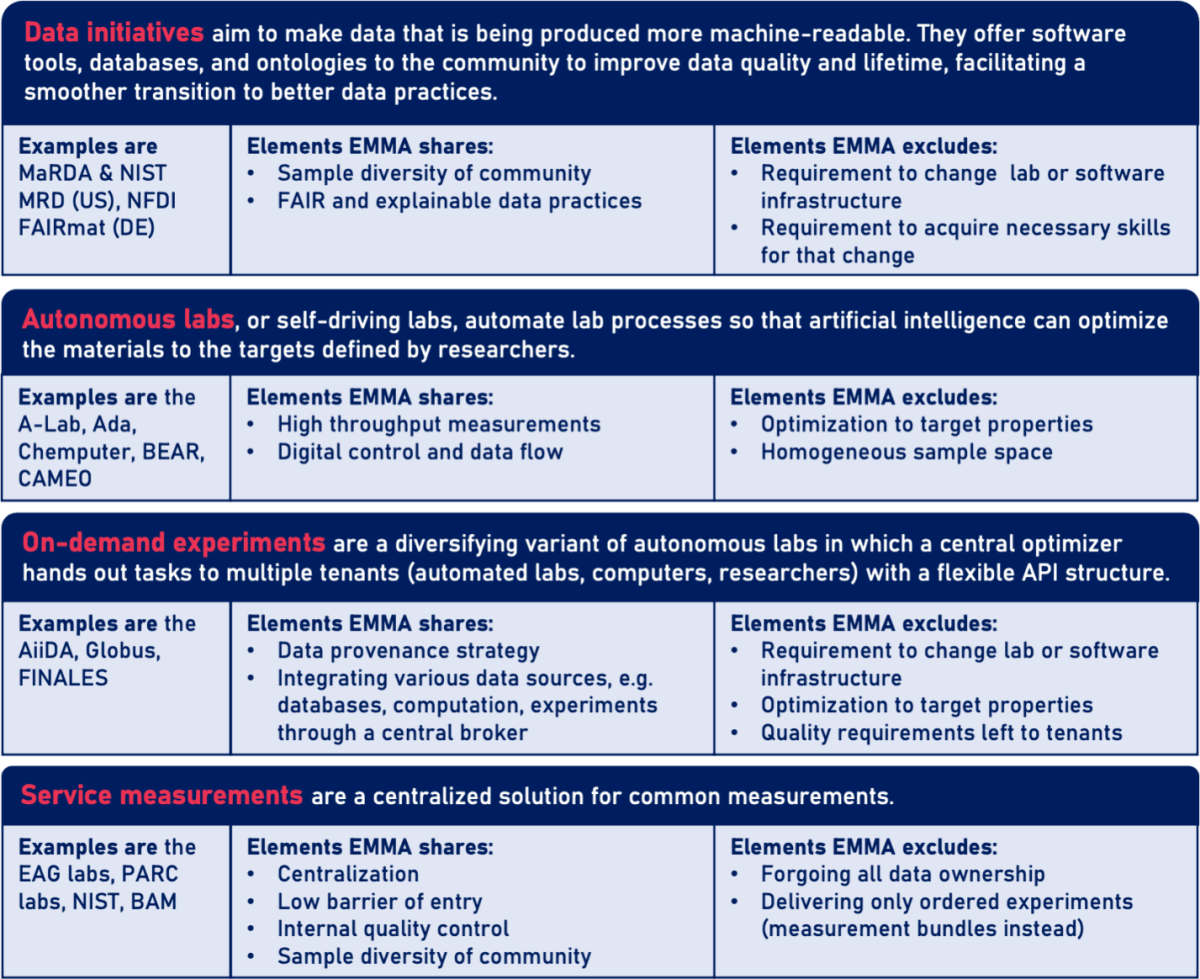
Main approaches to foster machine-readable data with examples; it is listed which elements of the approach are shared with the EMMA approach and which are purposefully excluded; references to the examples are given for MaRDA,^28^ NISTs MRD,^29^ NFDI FAIRmat,^30^ A-Lab,^31^ Ada,^32^ Chemputer,^33^ BEAR,^34^ CAMEO,^35^ AiiDA,^21^ Globus,^22^ and FINALES;^23^ it should be noted that, due to my personal background, the examples overrepresent the US and Germany.

Many approaches are already underway to address the goals defined in Table 1. The most prominent are given in Figure 3.

- Data initiatives foster FAIR data practices for materials research data in all its diversity. They develop examples and software tools to help the transition. However, changing the habits of thousands of researchers will take decades.
- Autonomous or self-driving laboratories put AI in the role of the researcher with the goal to optimize materials properties. They produce large amounts of data through automation, making them FAIR, explainable, and easy to produce, but are limited in their scope, have a large barrier of entry, and require continuity.
- On-demand experiments also use a central optimizer but tap into more diversity of research data by offering a flexible interface to a larger variety of experimental and computational setups that can be automated or performed by human researchers. The existing tools also foster data provenance but still suffer from either monotonous lab work or the cumbersome upgrade to digital control and data flow of the setup.
- Measurement services offer sample-to-data as a service. Their approach was not motivated by the promises of AI, but they offer a low barrier of entry for standardized measurements and low costs per measurement compared to the ownership of a setup. However, they have to limit their offerings to common measurement procedures to stay cost-effective.

**In this Perspective, I envision a centralized measurement service EMMA that combines elements of all the above approaches with the goal to reduce barriers of entry (make it easy), to cover a diverse range of samples, and to produce large amounts of FAIR and explainable data in a reasonable time frame.**

Figure 3 gives an overview of which elements of the above approaches are envisioned to be adapted and which are purposefully excluded from EMMA. In short, EMMA would increase sample diversity with respect to autonomous laboratories by measuring samples from the community as a service. It would adapt digital measurement control and data flow in a high-throughput manner, such as autonomous laboratories, and track data treatment (data provenance) for all integrated sources of data (databases, measurements, computation, etc.) such as that of the software infrastructure of on-demand experiments. The data and measurement quality is controlled internally, such as in services, and not left to tenants, as is the case for on-demand experiments. The optimization of material properties or functions are, however, left to the community and not to an internal AI as a service. The barrier of entry is low, since the measurements and the software and hardware integration are provided as a service and are not left to the community.

The resulting infrastructure would be well placed to serve a large part of the materials research community, overcome typical hurdles at shared costs, and produce large amounts of diverse yet FAIR and explainable data sets in a reasonable time frame. EMMA would thus be well placed to turbocharge the transition to accelerated materials discovery already underway.

What would this look like? Let us have a look behind the scenes of EMMA.

## One Morning at EMMA

*You take a clip from the charging wall, attach it to your neckline, and activate it. “Hi! Research service-agent 5 here, give me a report, please.” You hear a voice answering through the temples of your glasses:*

**Figure 4:**
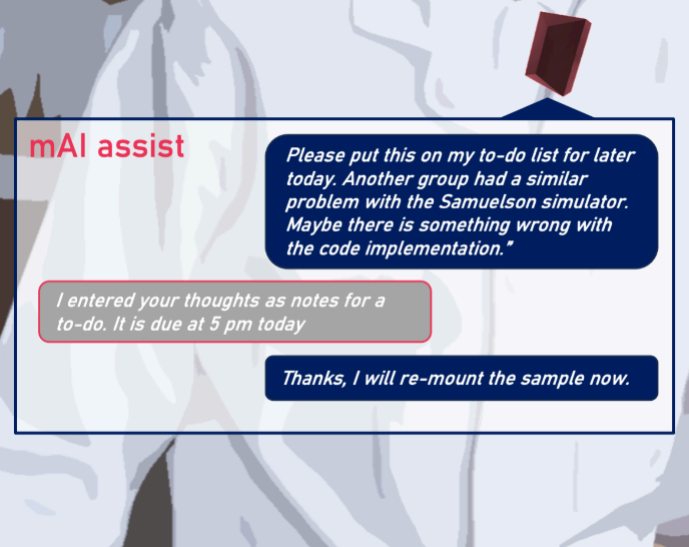
Illustration of the conversation between research service agents at the EMMA and the mAI assist clip.

*[mAI assist] (*Figure 4*) “Good morning and welcome back! Here is your report:*

- *Communications–you have 20 internal messages and 32 emails.*
- *Reviews–the XPS raw data correction algorithm by Werner et al. awaits your review before May 11. Your last note attached is “their definition of the sample orientation seems to be different from ours, so the sample-analyzer angle is off.”*
- *Bugs–issue 1, XRD temperature readings are missing since the firmware was updated on April 1; issue 2, the Samuelson X-ray scattering simulator needs adjustment for the upcoming density database API upgrade scheduled for August 4.*
- *Research services–the samples from Abuja and Kraków need your attention.”*

*[You] “Alright, I went running this morning and don’t feel like sitting down. Have me do lab work.”*

*[mAI assist] “I am glad you feel energized! Starting with the sample from Abuja. Please go to the transfer box in the bulk defect unit. The sample from Abuja is from the project named “Self-Healing Coating” and requested measurement sets for bulk defects and surface morphology. The sample needs to be remounted and transferred to the surface morphology unit. Air exposure is not a problem.”*

*[You] “OK! While I walk, please read the last email from this project.”*

*[mAI assist] “Sure! Nkiruka wrote to you on April 12: ‘My advisor and I decided to switch the X-ray scattering simulator because the Samuelson one did not give a satisfactory result with the usual optical constants. Does that make sense to you? We are looking forward to your opinion on that. Best, Nkiruka.’”*

*[You] “Please put this on my to-do list for later today. Another group had a similar problem with the Samuelson simulator. Maybe there is something wrong with the code implementation.”*

*[mAI assist] “I entered your thoughts as notes for a to-do. It is due at 5 pm today.”*

*[You] “Thanks, I will re-mount the sample now.”*

## The Key Features of EMMA

Admittedly, dreamy visions of the future of science tend to sound great, but the usefulness of the visions needs to be judged by the potential for change today. This is why the rest of this section will analyze the key elements of the vision and describe the way in which they advance over today’s approaches.

The four key features of EMMA are its centralized service infrastructure, measurement bundles, multivariate analyses, and the software ecosystem. Each is treated in a section with a short summary and its advances over today’s approaches.

### Centralized Service Infrastructure

The EMMA was portrayed as a centralized measurement service that receives samples from far and wide and measures them in a high throughput fashion. Time on EMMA’s instruments is, therefore, shared by customers.

The centralized services reduce the cost per measurement by the good utilization of scientific instruments and their efficient upkeep by experts. At the same time, it lowers financial risks for users and reduces their dependence on expertise within the local research group or institution. The same goes for the software infrastructure. Shared software and data management lowers the costs per user or data set.

The savings from centralized services can be reinvested in automation and in integration of software, instruments, and databases. The former lowers costs further, and the latter is one of the most daunting tasks for small research groups or institutions: software bugs, updated database interfaces, and digital interfaces to instruments all require continuous improvements of experts, a task difficult to realize with fixed-term contracts and fluctuating funding.

This integration of software, instruments, and databases would lead to more efficient research. Digitized instruments could deliver complete metadata, making measurements comparable, reproducible, and ultimately more trustworthy. Streamlining the data to a central depository also makes them FAIR (see Table 1). In effect, measurements do not need to be repeated and reproduced as often, again saving researchers time and money.

Not only are money and time saved but also centralized infrastructure can offer high-quality measurements and database services to more researchers. This is most valuable to the ones without access to high-end equipment, i.e., smaller universities or start-ups, but it also makes research more predictable for all, w.r.t. costs and time scales. The more widespread access ultimately increases the variety of samples and, thereby, measurement databases, enabling data science.

**In summary, a central service infrastructure offers a cost-effective way to produce large amounts of machine-readable data. In the medium and long-term, more automation could further lower the price and time per measurement. Wider accessibility to the services increases the variety of samples, while assuring data of high quality and long lifetime through integration of software, databases, and instruments. The four dimensions of big data, i.e., volume, variety, velocity, and veracity, can all be addressed with centralized service infrastructure.**

#### Advances over Today’s Approaches

Making experiments machine-readable poses barriers of entry in terms of cost, labor, and (re)education. Sharing the burden of overcoming these barriers via a centralized approach will help research groups in several ways: first, centralization would lower the barrier to produce FAIR and explainable data; second, it would produce software that can be reused in the field; third, it will reduce uncertainty about the usefulness of FAIR data, helping the decision whether to invest in it. Current services have evolved around answering specific questions, which require a more manual, customized approach. The service infrastructure suggested here would have a more data-centered focus. The associated loss of flexibility would be countered by more automation and hence a lower cost per measurement. This works especially well if the experiments are bundled.

### Measurement Bundles

In the “morning at the EMMA”, the measurements were organized in “units” for surface morphology or bulk defects. These units are automated measurement lines that group measurements by their usefulness for a research question. Surface morphology, for example, could contain techniques that provide useful information about the morphology and composition of the surface, such as GIXS (gracing incidence X-ray scattering), SEM (scanning electron microscopy), XPS (X-ray photoelectron spectroscopy), ellipsometry, and XRF (X-ray fluorescence).

The obvious benefit of making more measurements is ultimately to use the strengths of multiple techniques to reduce uncertainties in sample properties. Bundling them by research question is useful for practical reasons. Surface morphology, for example, might prefer flat and thin samples that can be mounted to the same type of sample holder and transported along the measurement line. Another benefit of bundling is the model to explain the sample (see Simulation-Based Multivariate Analyses below). Surface morphology is commonly described by nano/micrometric geometric structures that have average material properties. The same model would not work for techniques that can be explained only by atomic models, such as infrared spectroscopy or X-ray diffraction. Similarly, techniques that are used to answering similar hypotheses have significant overlap with the information obtained from them. This informational overlap should allow more robust interpretations than those from a set of techniques with little overlap.

Bundles also “encourage” negative experiments, which would not have been ordered as a service if they were known to fail. This is of benefit for the use of AI, since negative results also improve accuracy of prediction.^36^ An electrically isolating sample, for example, might charge during photoelectron spectroscopy and not produce evaluable results, but the physics of the experiment are still valid. If it is part of the training, then the prediction will be more accurate.

**In summary, measurement bundles are practical and can be automated for high throughput. The resulting sets are complementary, reinforce each other, and can be described by the same model. The bundles furthermore ensure that “useless”–but physically correct–measurements are preserved, supporting physics-based machine learning.**

#### Advances over Today’s Approaches

Measurement bundles as a service would create consistent and homogeneous data sets from multiple characterization techniques on a large variety of samples. Such a database does not exist currently: High-throughput laboratories are able to create large data sets but usually of quite homogeneous samples and with a limited number of characterization techniques; data initiatives naturally have diverse samples, but data are not yet consistent or homogeneous across multiple techniques. However, homogeneous databases and a pool of diverse samples are both required to make more general predictions from the data. Including “useless” measurements will further enhance the predictive power.

### Simulation-Based Multivariate Analyses

The student Nkiruka optimizes a central model that feeds into an array of measurement simulators (Figure 1). Their outputs are then compared against the results of all measurements in the measurement bundle. If the optical density of a material is changed, all results are affected.

**Figure 5:**
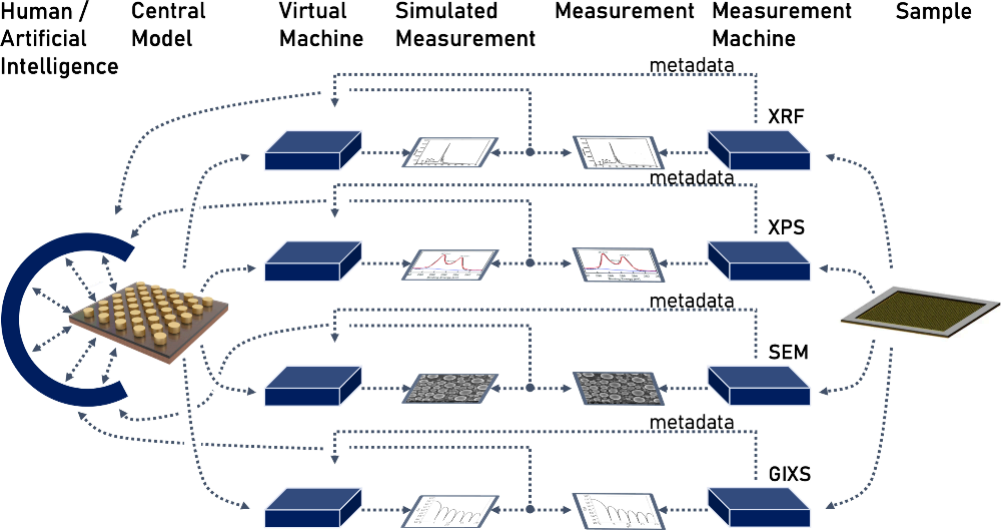
Flowchart of a multivariate analysis; a central model (left) is optimized by some form of intelligence to reduce the difference between simulated and measured results; for this to work, metadata describing the measurement needs to correctly enter the virtual measurement machine; each row in the figure is a straightened cycle from Figure 2; all of them are analyzed simultaneously; sample renderings in the figure are from the BornAgain software.

This is effectively a multivariate analysis (Figure 5), since it analyzes multiple variables simultaneously: exploring patterns rather than optimizing a single technique. Hence the name EMMA–Entity for Multivariate Material Analysis. Simultaneous analysis enforces consistency across all measurement techniques, making it more robust than the conventional sequential analysis that depends on the mind of the researcher to connect the dots correctly.

Multivariate analysis even goes beyond robust results: it makes material parameters determinable that would not be otherwise. Let us imagine a core–shell particle with a shell of unknown density and thickness. It is impossible to disentangle these two from one XPS spectrum or from one scattering pattern. If both are analyzed simultaneously, the thickness and density scale differently, and only one solution for both of them is viable. This goes for all parameters going into the model, making the analysis of unknown morphology more precise and robust, as it is known for the determination of nanostructure.^37^

When many central models have been optimized by researchers, further benefits can be reaped.

1. The first benefit is feeding materials properties from the optimized models back into databases for improving future predictions of them.
2. The second is using model parameters for data science. The statistical relationships between materials parameters can be used to find new predictive formulas and ultimately inspire faster measurement simulators.
3. The third is training AI to make a first guess on the central model from a set of measurements (mAI in the opening vision and Figure 5). The simulators ensure that the learning is physics based, and the databases provide statistical bounds for materials parameters, significantly narrowing the parameter space.
4. The fourth is finding exceptional materials, i.e., needle-in-a-haystack problems. True innovation happens when exceptional materials are found rather than materials adhering to statistical trends.^18,38^ The model databases of the centralized service are aimed at learning these statistical expectations, laying the groundwork for identifying exceptional material properties.

With the software solutions by EMMA (see also the following section on the Software Ecosystem), researchers would spend less time dealing with measuring, correcting, and analyzing data but instead focus on understanding the measurements and the model describing the sample (as in the opening vision). It takes a deep understanding of the sample and the underlying physics of the measurements to develop a fitting model, but the learning process would be more playful and intuitive and hence faster and more productive. The researchers can look at a 3D model with phase parameters and get feedback through simulated measurements (Figure 1).

**In summary, multivariate analyses create a self-consistent loop (**Figure 2**) connecting a sample and an interpretation model via measurements and respective measurement simulators. The optimized models can further be used to feed databases, to predict materials properties, and to train AI to predict models from measurements. Overall, this transformative workflow allows researchers to spend less time on data-related tasks and more time on understanding measurements, fostering faster learning and increased productivity.**

#### Advances over Today’s Approaches

Scientists have always used models to explain their findings. Similarly, digital twins are the backbones to store relevant data about a real world object.^39^ If realistic and complete enough, these digital twins are useful for materials scientists because they allow for physics-based machine learning. In their simplest form, material models are Wyckoff positions, symmetry, and a unit cell, as they are used in computational materials science. The open question is how more complex digital twins are created and updated based on a real-world object. Here is where the simulation-based multivariate analysis of experiments (Figure 5) could come to aid. The multivariate analysis is a traceable and explainable way to connect a model to a real-world object via comparison of measurement and simulation results. Within the error of simulation and experiment, multivariate analyses thus provide a metric for how well a model fits a real-world sample at the time of measurement. Think of creating a snapshot of a digital twin from measurements of a real-world sample (spot). The building stones of multimodal measurements, measurement simulations, and digital twins all exist today, but the advance of simulation-based multivariate analyses is to have an explainable and traceable way to create or update a digital twin. Applying it to the concept of the materials tetrahedron and its digital counterpart the materials-information twin tetrahedra (MITT, Deagen et al.^39^), simulation-based multivariate analysis as presented here could be seen as a way of connecting parts of the two tetrahedra living in the real and digital world.

### Software Ecosystem

**Figure 6:**
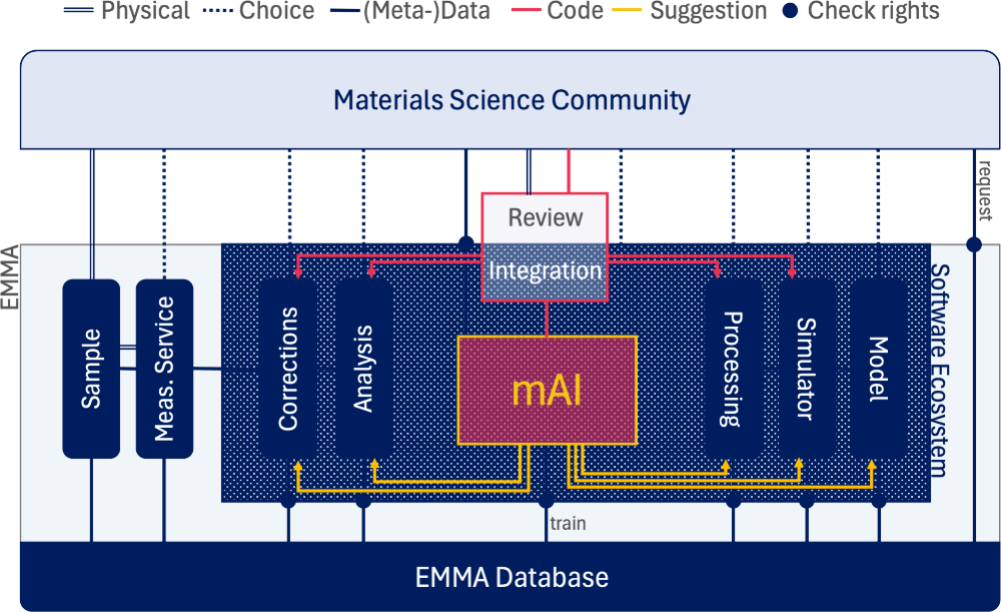
Flow of information between the materials science community (top) and EMMA (bottom); the dark blue columns are equivalent to the elements in the sample characterization cycle of Figure 2; the community sends samples to EMMA, where they are measured as a service; raw sample and measurement metadata are saved in the EMMA database before they enter the software ecosystem (dark blue area); in the multivariate analysis, the researcher from the community picks the algorithms for data correction, analysis, experiment simulation, and processing of outputs and optimizes the model; optimized model parameters and code versions are saved in the database; the process is supported by suggestions from mAI, which trains on the database; new methods in the form of code are suggested and reviewed by the community and integrated into the software ecosystem of the EMMA after approval; data protection is assured by strict user rights, including AI algorithms that train on the database.

In the vision, the research service agent had to review a new data correction algorithm for publication and integration into the digital services of the EMMA (compare Figure 6). Another task was bug-fixing–integrating a thermal reading or adjusting the software interface between a database and a simulator. The digital services of the EMMA are portrayed as a software ecosystem that constantly evolves through feedback, improvements, and new code.

Such an integrated software ecosystem can be powerful because it is developed and improved by a large community and made user-friendly by experts at EMMA interfacing and integrating it with other pieces of software and databases (see Figure 6 for a flow of information). New methods are submitted as a manuscript and code, reviewed, published, integrated, versioned, chosen by users in a drop-down menu (Figure 1), critiqued, updated, and maintained. This process-oriented system can make new ways of treating data more accessible to researchers for longer.

When results are published, all steps of the process from raw data to model (Figure 2) are published along. This open process of data treatment by versioned code (data and code flow in Figure 6) assures transparency and allows critique, which ultimately improves the accuracy of results and trust in them. Manuscript reviewers could check how data was treated and if it was appropriate to obtain the claimed results. They could even get collaborative access and suggest a new branch of analysis that is more correct.

Another benefit of integrated software and data structures is the compatibility with new methods for a longer time. Data that were recorded years ago can be revisited with improved simulators, more appropriate data correction algorithms, or updated databases. Old data keeps improving or can be made more comparable to new results, increasing the long-term value of data.

**In summary, the software ecosystem envisioned for EMMA is process-oriented and undergoes continuous refinement. New algorithms are incorporated through a publication-like system; bugs are fixed, and software interfaces are adjusted by experts. The transparent and open process involves the submission, review, publication, integration, and versioning of data treatment methods, making them more accessible to nonexpert researchers. This ecosystem can lead to more ease of use, transparency, and trust in results and enhances the long-term value of data.**

#### Advances over Today’s Approaches

Research software engineering, or RSE, is already recognized as a crucial building block for the future of science. Computational materials science projects like AiiDA or Globus, pioneered software ecosystems, include data provenance (tracking data treatment) from the model to the result. AiiDA also allows community contributions, which has been used to apply it on experiments as well.^24^ For experimentation, similar software tools are bluesky,^40^ ESCALATE,^41^ and ChemOS.^42^ The advances of the ecosystem in Figure 6 are thus adapting the above projects for a range of experimental setups and software as well as institutionalizing the process of code contributions and their integration.

## Foreseeable Challenges

The centralized infrastructure, measurement bundles, multivariate analysis, and software ecosystem combined offer many benefits that promise to accelerate the transition to data-centered materials science. However, the approach involves trade-offs that could turn into challenges. The following paragraphs list three conceptual challenges that go beyond practical issues such as initial investment, continuous funding, or high-maintenance software/instrumentation.

First is centralization. If EMMA were an integral part of the materials science infrastructure, corporate and academic research could start depending on it for the realization of their research goals. Such a systemic relevance comes with responsibility and accountability that need to be foreseen in the EMMAs legal framework from the start. A nonprofit status and independent oversight would be a sensible starting point.

Second is the intellectual property. In the EMMA scenario, the community would send samples for service measurements and contribute code for data processing or simulation. However, the data and software would be hosted centrally, raising the question of ownership that must be addressed. In an open science setting, this would not be a problem, but competition of any form could hamper that approach. A sensible starting point would be to put a price on the extent of a data embargo (e.g., no data use, only AI training, open data use) and its duration (e.g., any embargo longer than 2 years; renewal every 5 years or open data use otherwise).

Third is a partial research focus. EMMA focuses on characterization and purposefully excludes synthesis and functional characterization. This focus has the beneficial aspects that it creates an unbiased part of the infrastructure that supports ongoing research but risks a hidden connection between properties and function. This hidden connection would alleviate data ownership issues but could also hamper a more generalized prediction of function property relationships. A sensible starting point would be a mandatory unique identifier for any EMMA data set published in part or as a whole, with the goal to ease data mining for the function of materials. Another alleviating factor in this context is that operando structure might anyway not be the same as ex situ structure but also limits the scope of EMMA.

## Conclusion

I have envisaged EMMA, an Entity for Multivariate Material Analysis, offering measurement bundles to address common research needs in materials science. The centralized approach enables EMMA to produce extensive metadata to make raw data explainable, FAIR (findable, accessible, interoperable, reusable), and machine-readable.

A key aspect of EMMA is a simulation-based multivariate analysis of these measurement bundles, where measurement simulators are fed by a central model and produce results that are compared against all measurements in the bundle. This simultaneous optimization of central model parameters ensures robust, user-friendly, and explainable analyses. The resulting digital model can serve as a data source for understanding materials properties and how they are connected.

This homogeneous and yet diverse experiment database is integrated into a peer-reviewed software ecosystem. All code altering raw data, spanning from its inception to the final result, is submitted, reviewed, versioned, and integrated into the database by experts at the EMMA. This process-oriented ecosystem improves transparency, reduces uncertainty of results, and extends the data’s usefulness, strengthening important pillars of science, i.e., reproducibility, transparency, consistency, accuracy, and falsifiability.

Data science would thrive on this combination of high-quality experimental data sets, digital models, and integrated code. The databases could enable tasks such as finding new predictive formulas, training AI, and benchmarking computational methods. EMMA’s services and innovations aim at streamlining processes, freeing up time and resources for human researchers to excel in information collection, questioning, hypothesis generation, and experiment design.

As we urgently need AI to accelerate the innovation of energy materials, we need to rapidly decrease barriers to produce large amounts of high quality data that can be understood by machines. Only then can we make the most of AI and free up resources to accelerate material innovation. A service infrastructure such as the envisioned EMMA could do that for well-established experiments and common research needs.

## Outlook

The vision for EMMA is now laid out, but which steps need to be taken to approach this vision are left open.

A necessary first step is to scientifically test whether the envisioned advances discussed above can provide justifications for investment in the approach. We saw that the simulation-based multivariate analysis is the largest advance compared to current procedures, but thorough scientific testing is needed. If simulation-based multivariate analyses can indeed connect a sample with a model representation and be partially automated by machine learning, then the value of the approach justifies investment. Another facet that needs to be quantified is the benefit of centralized services. If it can be demonstrated that centralization lowers the cost while producing data of higher quality, investment is further justified.

Given that the investment is justified, the second step is to implement a service laboratory on a local scale. A university or cluster of universities could invest in central nonprofit service laboratories with a focus on FAIR data production. In this framework, software solutions and measurement automation could be tried, developed, and integrated with research needs of the university (cluster) in mind.

Based on the experience of the local initiative, the third step is to institutionalize the approach further and to build a first version of the EMMA. With regard to the software infrastructure, the institution could follow the example of the Red Hat Inc. that initialized open source software, and combine that approach with that of scientific journals. The advanced materials lab infrastructure and sample logistics of EAG laboratories as well as the automation and digital integration of the Emerald Cloud Lab could serve as examples to develop the physical infrastructure.

To illustrate how a measurement service like the EMMA could indeed start from a local initiative, let me share an inspiring story from Berlin. In 2011, the clinics of Berlin came together to establish Labor Berlin, a centralized medical service lab. Fast forward to today, Labor Berlin is a high-throughput lab receiving 20,000 samples every day from across Germany and Europe. It has become a distinguished innovator of methods and laboratory processes, showcasing how a centralized service solution can be an excellent way to cut costs and drive innovation.

Although materials science laboratories are different from medical laboratories, both fields have common research needs that established methods can address. Centralized services can effectively cater to these requirements and accelerate innovation within them. Envision a local initiative that integrates software and measurement services and grows into a hub for data-driven materials innovation. Would you like to join?
